# The Role of Electrostatic Repulsion on Increasing Surface Activity of Anionic Surfactants in the Presence of Hydrophilic Silica Nanoparticles

**DOI:** 10.1038/s41598-018-25493-7

**Published:** 2018-05-08

**Authors:** Hamid Vatanparast, Farshid Shahabi, Alireza Bahramian, Aliyar Javadi, Reinhard Miller

**Affiliations:** 1 Institute of Petroleum Engineering, College of Engineering, University of Tehran, Tehran, Iran; 2 IOR Research Institute (IORI), Tehran, Iran; 3 Max-Planck-Institute for Colloid and Interface Science, D-14476 Golm, Germany

## Abstract

Hydrophilic silica nanoparticles alone are not surface active. They, however, develop a strong electrostatic interaction with ionic surfactants and consequently affect their surface behavior. We report the interfacial behavior of n-heptane/anionic-surfactant-solutions in the presence of hydrophilic silica nanoparticles. The surfactants are sodium dodecyl sulfate (SDS) and dodecyl benzene sulfonic acid (DBSA), and the diameters of the used particles are 9 and 30 nm. Using experimental tensiometry, we show that nanoparticles retain their non-surface-active nature in the presence of surfactants and the surface activity of surfactant directly increases with the concentration of nanoparticles. This fact was attributed to the electrostatic repulsive interaction between the negatively charged nanoparticles and the anionic surfactant molecules. The role of electrostatic repulsion on increasing surface activity of the surfactant has been discussed. Further investigations have been performed for screening the double layer charge of the nanoparticles in the presence of salt. Moreover, the hydrolysis of SDS molecules in the presence of silica nanoparticles and the interaction of nanoparticles with SDS inherent impurities have been studied. According to our experimental observations, silica nanoparticles alleviate the effects of dodecanol, formed by SDS hydrolysis, on the interfacial properties of SDS solution.

## Introduction

Surfactants are extremely versatile chemical products of the chemical industry. They appear almost everywhere and in diverse products like detergents, pharmaceuticals, wettability modifiers, drilling muds, motor oils, and even foods. Presence of nanoparticles, which is unavoidable in many industrial activities such as chemical enhanced oil recovery (EOR) processes, can considerably affect the behavior of surfactants^1–4^. The complex system of surfactant-nanoparticle functions as a novel surface-modifying agent and can be used for stabilizing disperse systems such as foams and emulsions^5–8^. The role of surfactant-nanoparticle interactions on the fluid/fluid interfacial behavior, however, is still not clearly known and is currently a topic of increasing interest^9^. In the presence of ionic surfactants, the electrostatic attractions and repulsions increase the complexity of interfacial behavior. Generally, untreated nanoparticles such as hydrophilic silica are not surface active but their presence in a system containing surfactants may strongly affect the interfacial behavior of the liquid^10,11^. A great number of valuable studies have been performed with regard to the interfacial behavior of surfactant-nanoparticle systems, but mostly focused on the oppositely-charged surfactant-particle systems (e.g. silica nanoparticles and cationic surfactant of CTAB^10,12–28^). According to these studies, the electrostatic attraction between the negatively charged surface of the silica particles and the oppositely charged cationic surfactant molecules promotes the surfactant adsorption at the nanoparticles’ surfaces. It is now well accepted that the partial modification of the hydrophilic/hydrophobic character by the surfactant adsorption is the main reason for driving nanoparticles to liquid interfaces^24,25^. In this case, surface-modified nanoparticles function as novel surface-modifying agents and increase the stability of colloidal systems like foams and emulsions by improving their interfacial rheological properties^8,21,26–28^.

In contrast to the cationic surfactants, anionic surfactants do not adsorb at the surface of silica nanoparticles. Therefore, the *in-situ* modification of nanoparticles’ surfaces and the formation of surface-active complexes of surfactant-nanoparticle are not expected. This is probably the reason why much less attention has been paid to studies of the surface/interfacial behavior of systems comprised of surfactants and nanoparticles with the same electric charge. However, the results of the few studies performed in this regard imply that the nanoparticles can influence the surface activity of a similarly charged surfactant through electrostatic repulsive interaction^29–33^. According to these results, the surface/interfacial tension of a SDS solution, below its critical micelle concentration, is lower in the presence of negatively charged SiO_2_ or ZrO_2_ nanoparticles.

We have recently^33^ investigated the effect of hydrophilic silica nanoparticles on the surface properties of SDS solutions by measuring the dynamic surface tension, the dynamic foamability, the stability of the established foams, and also the low-frequency surface rheology. Moreover, the surface adsorption of nanoparticles was studied by applying high amplitude compressions/expansions of a drop surface. We found that for systems containing nanoparticles, SDS equivalently behaved as if higher concentrations of SDS were used. The results also indicated that the dynamics of adsorption and the response of the interfacial layer to the surface perturbations were faster for systems containing nanoparticles. These observations imply the enhanced surface activity of surfactant in the presence of similarly charged hydrophilic silica particles.

In the present paper, we have focused on the interfacial behavior of similar systems at liquid/liquid interfaces. The selected anionic surfactants are SDS and DBSA, and the selected particle is nano-sized silica with different sizes of 9 and 30 nm. A systematic study based on the profile analysis tensiometry and a further supplementary discussion have been provided to demonstrate the role of electrostatic repulsion in the variation of surfactant’s surface activity. We have used different salt concentrations to change the surface charge of the nanoparticles and to control the repulsion between nanoparticles and surfactant molecules. Furthermore, to clarify the fast dynamics of the surfactant absorption in the presence of nanoparticles, the hydrolysis of SDS molecules in the presence of nanoparticles and the interaction of silica nanoparticles with SDS inherent impurities have also been investigated.

## Results and Discussion

### Surface activity of pure nanoparticles

The interfacial tension and interfacial elasticity between various concentrations of dispersed silica nanoparticles (Levasil 300/30, 9 nm) and n-heptane was found to be close to those between n-heptane and pure water (Figs S1 and S2). Thus, these hydrophilic nanoparticles are not surface active. Similar results have been obtained for another nanofluid (Levasil 100/45, 30 nm) which confirms these untreated nanoparticles have no surface-active character. Therefore, in the systems of nanoparticles mixed with surfactant, the deviations of interfacial properties from those of the pure surfactant system could be reasonably attributed to nanoparticles-surfactant interactions.

### Effect of nanoparticles on the interfacial tension of SDS solutions

**Figure 1:**
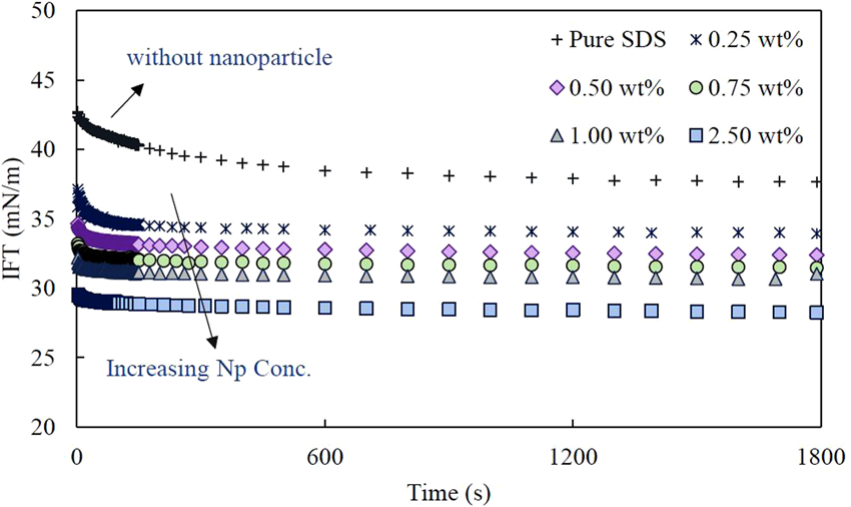
Dynamic interfacial tension of n-heptane/SDS solutions in the presence of different amounts of nanoparticles (Levasil 300/30, 9 nm). The surfactant concentration was fixed at 4.1 × 10^−1^ mM (0.05 CMC) in all experiments. The average error for each experiment is less than 0.5 mN/m.

The dynamic interfacial tension of n-heptane/water containing SDS at a constant concentration (4.1 × 10^−2^ mM; 0.05 CMC) and different concentrations of nanoparticles (Levasil 300/30, 9 nm) are presented in Fig.1. In these experiments, the nanoparticle concentration varied from 0 to 2.5 wt. %. As shown in this figure, when no nanoparticles exist in the system, the IFT reduces quickly, valued initially at about 42 mN/m. However, it gradually approaches the equilibrium value within more than 600 s. For the mixed surfactant-nanoparticle systems, both the starting point and the equilibrium values of IFTs are lower. Moreover, the dynamics of adsorption is faster and the IFT reaches the equilibrium values in a shorter time with increasing nanoparticle concentration (less than 100 s for all cases).

We have shown previously for CTAB-silica nanoparticle systems^34^, where nanoparticles adsorb at the interface (along with surfactant), that the dynamics of adsorption is slower than that of the pure surfactant system. In such case, the IFT starts from a higher value, close to the pure water/heptane IFT, and finally reaches a significantly lower equilibrium value. The dynamic IFT change, however, is slow. The reason is the smaller diffusion coefficient of surfactant-nanoparticle complexes due to their much larger sizes in comparison with the surfactant molecules. Therefore, it can be stated that the faster dynamics of adsorption, observed in Fig. 1, indicate a negligible or no adsorption of silica nanoparticles at the interface in mixed systems of SDS-nanoparticles. In other words, nanoparticles still retain their non-surface-active nature. The results of zeta potential measurements also reject the modification of nanoparticles’ surface charges by the surfactant molecules. The zeta potential of nanoparticles in the diluted nanofluid dispersion (0.25 wt.%) almost remained unchanged by adding 0.41 mM SDS (−27.4 ± 1 mV). This result was quite expectable considering the same electrical charge of the nanoparticles’ surface and the surfactants’ head group. However, in the presence of nanoparticles the surfactant behaves more efficient in reducing the IFT. This behavior can be attributed to the electrostatic repulsion between SDS molecules and nanoparticles which promotes the surfactant adsorption.

### Effect of nanoparticles on the interfacial elasticity of SDS solutions

**Figure 2:**
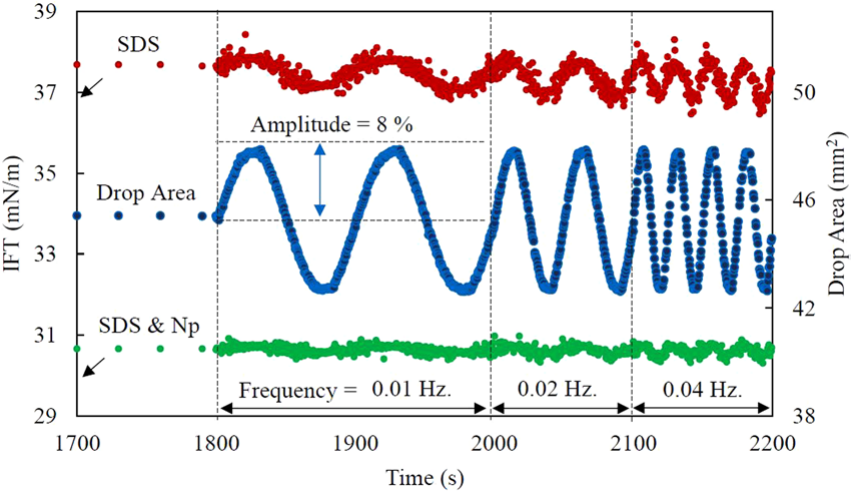
IFT variations during sinusoidal drop oscillation for an SDS solution (0.05 CMC) without (upper curve) and with (lower curve) 1.0 wt.% nanoparticle (Levasil 300/30, 9 nm). The middle curve shows drop surface area variations during oscillation.

Figure 2 shows IFT variations during sinusoidal oscillation of the pre-equilibrated interface at three different perturbations frequencies for two SDS solutions, without and with 1 wt.% of nanoparticles (Levasil 300/30, 9 nm). By applying the same drop surface changes, the IFT of both systems changes slightly. However, in the presence of nanoparticles, the interfacial responses to the surfactant adsorption and desorption are faster. The variation of IFT for the SDS-only system is about 1.0 mN/m while it is negligible for the system containing nanoparticles.

The interfacial elasticity modulus for all the studied systems were calculated using the IFT variations during drop surface oscillation. According to the obtained results (Fig. S3), the surfactant solution has an elasticity of 6 and 9 mN/m depending on the oscillation frequency (between 0.01 and 0.1 Hz.). It is worth mentioning that at the applied low-frequency range of perturbations, the obtained elasticity values for SDS solutions cannot be considered as the maximum elasticity values. However, it helps to evaluate the trend of elasticity variation by the addition of nanoparticles.

In the presence of 0.25 wt.% nanoparticles, the elasticity values are much lower than the SDS-only solutions (2.8 and 4.8 mN/m at the oscillation frequency of 0.01 and 0.1 Hz., respectively) and it decreases slightly with increasing nanoparticle concentration. These results are well consistent with the fast dynamics of the IFT reduction for the mixed SDS-nanoparticle dispersions. Moreover, considering the low values of the elasticity modulus in the presence of nanoparticles and their relative values compared to that of SDS-only solutions, it can be reasonably stated that nanoparticles do not adsorb at the interface. As it has been shown in the previous studies^12,13,34^, the interfacial adsorption of nanoparticles results in high elasticity values which increase with increasing the concentration of nanoparticles. In addition, the interfacial response of the nanoparticle-contained mixture to the surface perturbation cannot be as fast as that of the surfactant-only solutions.

### Interfacial behavior at large drop surface area changes

**Figure 3:**
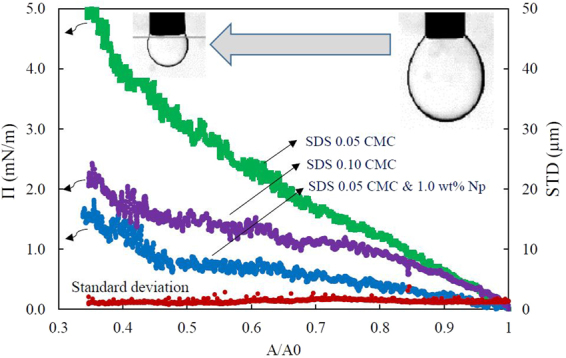
Variation of surface pressure and standard deviation of fitting the Laplace equation during drop compression of two SDS solutions (0.05 and 0.10 CMC) and SDS solution (0.05 CMC) mixed with 1 wt.% nanoparticle (Levasil 300/30, 9 nm). Π: surface pressure, STD: the standard deviation of fitting the Laplace equation, A/A0: the normalized surface area of droplet in respect to its initial value. The average error per point for surface pressure results is less than 0.3 mN/m and for standard deviation results is insignificant.

More detailed study of the adsorption of nanoparticles at the heptane/water interface has been carried out by performing large amplitude compressions of the pre-equilibrated drop surface and measuring the response of the interfacial layer to these perturbations. Figure 3 shows the variation of surface pressure, Π (IFT difference from equilibrium condition), and the standard deviation of fitting the Laplace equation, STD, during drop compression of three systems containing (1) 0.05 CMC SDS, (2) 0.10 CMC SDS, and (3) 0.05 CMC SDS plus 1 wt.% silica nanoparticles (Levasil 300/30, 9 nm). As it is shown in Fig. 3, by reducing the drop surface, the surface pressure for SDS solution at the concentration of 0.05 CMC gradually increases and reaches the value of about 5 mN/m at the maximum applied compression. However, at the same SDS concentration, the surface pressure variation is lower for the system containing nanoparticles. The standard deviation of fitting the Laplace equation is almost constant and nearly zero during the compression of the interface for all the three systems. It means that the drop retains its Laplacian shape even at the maximum surface compression.

Both the overall trend of changes in the surface pressure and the standard deviation of fitting the Laplace equation indicate that nanoparticles do not adsorb at the interface. As we have shown previously^33,34^, both liquid/liquid and air/liquid interfaces behave differently when nanoparticles lie in the drop surface. In this case, the surface pressure value and its rate of changes, are higher over the course of interfacial compression. The surface pressure increases until it reaches a plateau where the maximum packing of particles at the interface occurs, after which the surface pressure increases sharply indicating the collapse of the nanoparticles monolayer formed on the drop surface. There is also a significant deviation of the drop shape from its Laplacian shape when the drop surface is covered by nanoparticle at the maximum compression. The standard deviation abruptly rises at the onset of the closed-packed state of nanoparticles at the interface and during the collapse of the nanoparticle layer. Therefore, the lower interfacial tension of SDS-Silica nanoparticle systems can be just attributed to the higher surface activity of SDS molecules in the presence of silica particles and not to the interfacial adsorption of particles. The surfactant molecules in the presence of nanoparticles, thus, are more efficient, as if a higher concentration is used.

### Equivalent surfactant concentration (EC) of composite surfactant-nanoparticle systems

**Figure 4:**
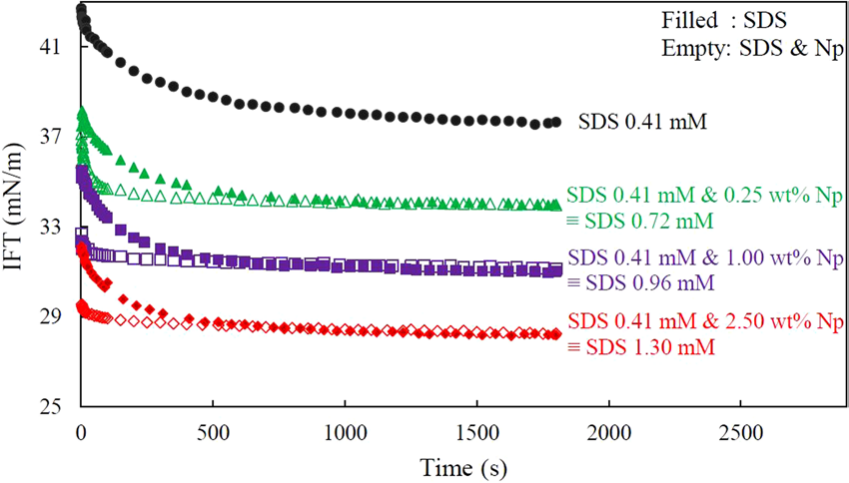
Estimation of the equivalent surfactant concentration (EC) of mixed surfactant-particle solutions by matching their equilibrium IFTs with those of surfactant-only solutions.

The increase of surfactant surface activity in the presence of nanoparticles (Levasil 300/30, 9 nm) has been quantified and stated as the equivalent surfactant concentration (EC). First, the dynamic IFTs of pure surfactant solutions were measured in a wide range of surfactant concentration. Then, the equilibrium IFT values were used as a criterion for comparing the mixed SDS-nanoparticles systems with pure surfactant solutions. According to the results, there is a linear trend between nanoparticle concentration and the EC values of the systems (Fig. S4) which indicates that the surface activity of surfactant linearly increases with the concentration of nanoparticles. However, the dynamic IFTs of the mixed SDS-nanoparticle solutions are different from the dynamic IFTs of the SDS-only solutions of the corresponding EC concentrations. As it is shown in Fig. 4, in the presence of the nanoparticles, the dynamics of adsorption is fast and for two systems with identical equilibrium IFT, the systems containing nanoparticles (empty symbols) have much faster dynamics compared with the dynamics of the pure surfactant system (filled symbols).

### Effect of nanoparticles on SDS surface activity in presence of salt

**Figure 5:**
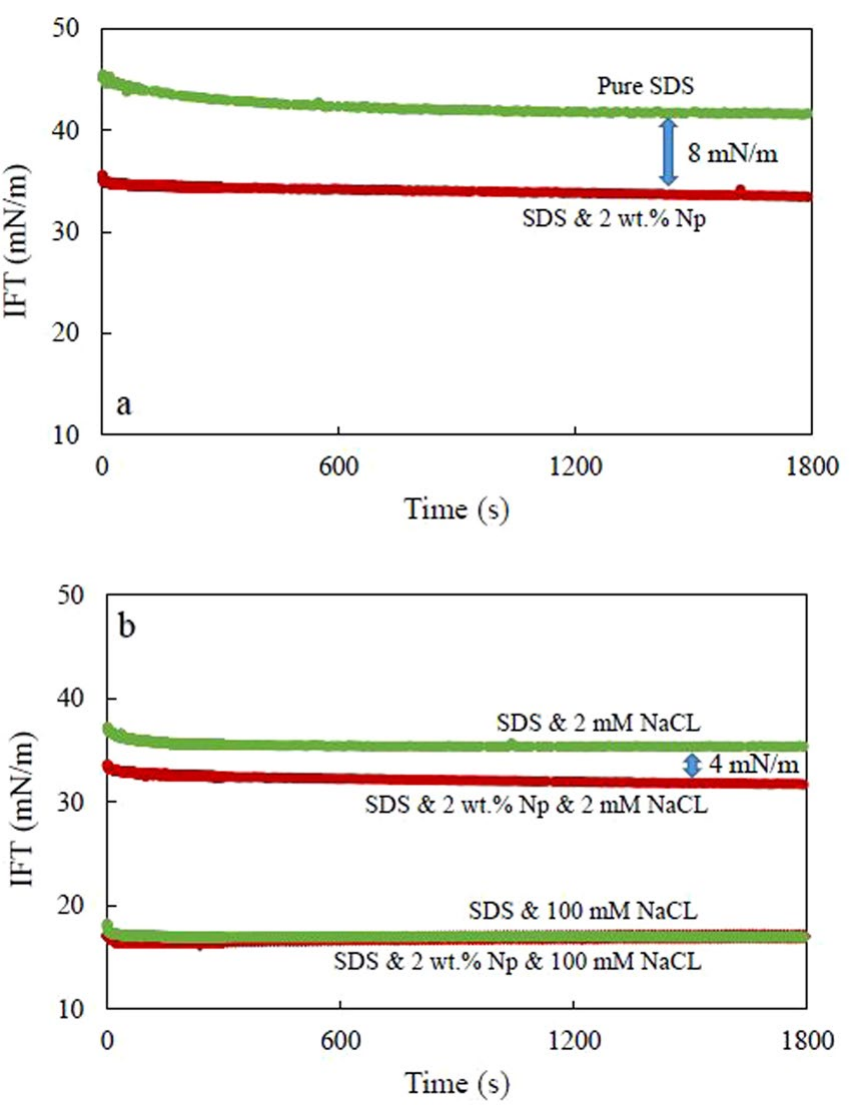
The effect of nanoparticles (Levasil 100/45, 30 nm) on dynamic IFT of heptane/SDS solution (**a**) without salt, (**b**) in the presence of NaCl at concentrations of 2 and 100 mM. In all experiments, the surfactant and nanoparticle concentrations were fixed at 2.9 × 10^−1^ mM (0.035 CMC) and 2 wt.%, respectively. The average error for each experiment is less than 0.5 mN/m.

The observed results of the effect of silica nanoparticles on the interfacial behavior of anionic surfactant have also been evaluated by measuring dynamic IFTs of SDS solutions in the presence of another sample of silica nanoparticles. Moreover, the role of the electrostatic repulsion in increasing the surfactant surface activity has been further explored in the presence of salt which can screen both nanoparticle and surfactant surface charges. Figure 5a shows the impact of silica nanoparticles with a larger size (Levasil 100/45, 30 nm) on the dynamic IFT of SDS solution at a concentration of 2.9 × 10^−1^ mM (0.035 CMC). As it can be seen from this figure, the dynamic IFT of the mixed SDS-nanoparticle solution is much faster and about 8 mN/m lower than the IFT values of the SDS-only solution.

Similar experiments have been conducted in the presence of NaCl. As shown in Fig. 5b, SDS solution has lower IFTs in the salt solutions, as it is the characteristic of ionic surfactant systems^35–37^. The impact of nanoparticles on the interfacial tension of the surfactant solution weakens in the presence of NaCl and it completely vanishes at high concentrations. The IFT difference arising from the presence of nanoparticles in the systems decreased to about 4 mN/m when 2 mM NaCl is added and fades away at salt concentration of 100 mM.

**Figure 6:**
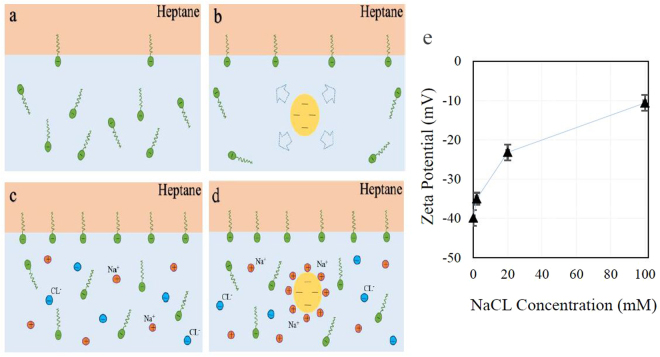
(**a**–**d**) Schematic presentation of the impact of the repulsive interaction between nanoparticles and surfactants to enhance the surfactant’s surface activity. (**a**,**b**) without salt, (**c**,**d**) in the presence of NaCl. (**e**) Variation of zeta potential of 2 wt.% nanofluid (Levasil 100/45, 30 nm) with the concentration of NaCL. The line is drawn as guide for the eyes.

These results support the validity of the proposed theory of increasing surfactant surface activity in the presence of repulsive nanoparticles (schematically shown in Fig. 6a and b). The addition of electrolyte promotes the surfactant activity coefficient and accordingly increases its surface activity^35,38^ (Fig. 6c). However, it can also shield similarly charged surfactants and nanoparticles from the electrostatic repulsion. Therefore, at the high concentration of salt, the electrostatic repulsion almost disappear due to the entire surface screening of the negatively charged nanoparticles’ by Na^+^ ions (Fig. 6d). The measured Zeta potential values of the nanoparticles in the salt solutions, shown in Fig. 6e, supports the impact of salts in reducing the electrostatic forces between surfactant and particles.

To quantify the amount of surface activity variations by the addition of nanoparticles, we used the Frumkin model to interpret our measured isotherms. Frumkin adsorption isotherm is a three-parameter model which considers the intermolecular interaction between the adsorbed surfactant molecules in the monolayer and relates the interfacial tensions (${\gamma }_{0}\& \,\gamma$) to the bulk surfactant concentration (c) and to the surface coverage ($\theta \,=\,\Gamma /{\Gamma }_{\infty }$) as^39,40^:

1 $$\begin{array}{cccc}{bc} & = & \frac{\theta }{1-\theta }{\exp }(-2\alpha \theta ) & \,\mathrm{Equation}\,\mathrm{of}\,\mathrm{state}\end{array}$$

2 $$\begin{array}{cccc}\gamma  & = & {\gamma }_{0}\,+\,{RT}{\Gamma }_{\infty }[{\rm{ln}}(1-\theta )\,+\,\alpha {\theta }^{2}] & \,\mathrm{Adsorption}\,\mathrm{isotherm}\end{array}$$

where R is the gas law constant, T is the temperature, b is the adsorption equilibrium constant, $\Gamma$ and ${\Gamma }_{{\rm{\infty }}}$ are the actual and the saturation adsorptions, and $\alpha$ is the interaction parameter representing the lateral interaction energy of the adsorbed surfactant molecules. For ionic surfactants $\alpha$ has two terms: the attraction interaction between the surfactants hydrophobic chains and the electrostatic repulsion interaction between their polar head-groups^41–43^. According to Frumkin model, the positive and negative values of $\alpha$ reflect the net attraction and repulsion interaction, respectively. In the case that there is no interaction between the adsorbed surfactant molecules, the Frumkin model reduces to the Langmuir model.

**Figure 7:**
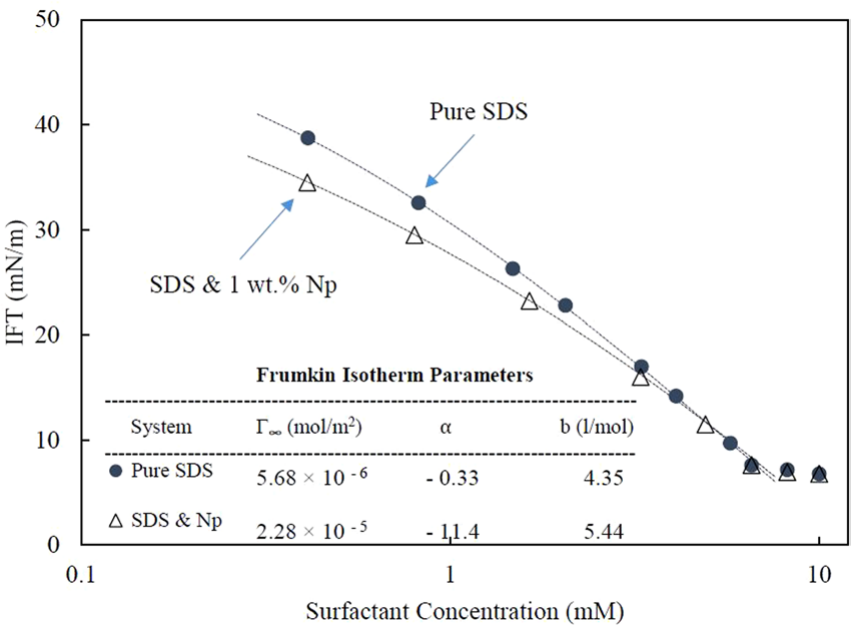
The effect of nanoparticles (Levasil 100/45, 30 nm, 1 wt.%) on equilibrium interfacial tension of heptane/SDS solution as a function of SDS concentration. The dotted lines represent the Frumkin isotherms with the fitting parameters shown in the figure (Γ: adsorption, α: intermolecular interactions and b: adsorption equilibrium constant). The average error for each experiment is less than 0.5 mN/m.

Figure 7 shows the equilibrium IFT as a function of SDS concentration for SDS-only and SDS-nanoparticle (Levasil 100/45, 30 nm) solutions. The best-fit of Frumkin isotherms with the fitting parameters have also been shown in the figure. According to the obtained results, in the presence of nanoparticles the adsorption value (${\Gamma }_{{\rm{\infty }}}$) has increased from 5.68 to 22.8 µmol/m^2^. The best fit value of the interaction parameter is almost negligible ($\alpha =-0.33$) for pure surfactant system. The negative value, however, indicates the repulsion between surfactant molecules at the interface. For the mixed surfactant-nanoparticles system, $\alpha$ is −11.4, implying significant repulsive interactions between the surfactant molecules adsorbed at water/heptane interface.

At low SDS concentrations, the repulsive interaction between nanoparticles and surfactant molecules results in the more adsorption of surfactants and subsequently the higher repulsive interaction between the adsorbed surfactant molecules ($\alpha$), resulting from their closer orientation at the interface. However, as shown in the Fig. 7, the impact of nanoparticles weakens by increasing in the surfactant concentration and almost disappears at surfactant CMC and higher concentrations, where the negatively charged micelles outnumber the particles. Moreover, at the higher SDS concentration, the Na^+^ ions dissociated from the SDS structure (C_12_H_25_SO_4_^−^ Na^+^) effectively screen the negatively charged nanoparticles and weaken the repulsive interaction between surfactant and nanoparticle.

### Effect of negatively charged silica nanoparticle on the interfacial behavior of DBSA

**Figure 8:**
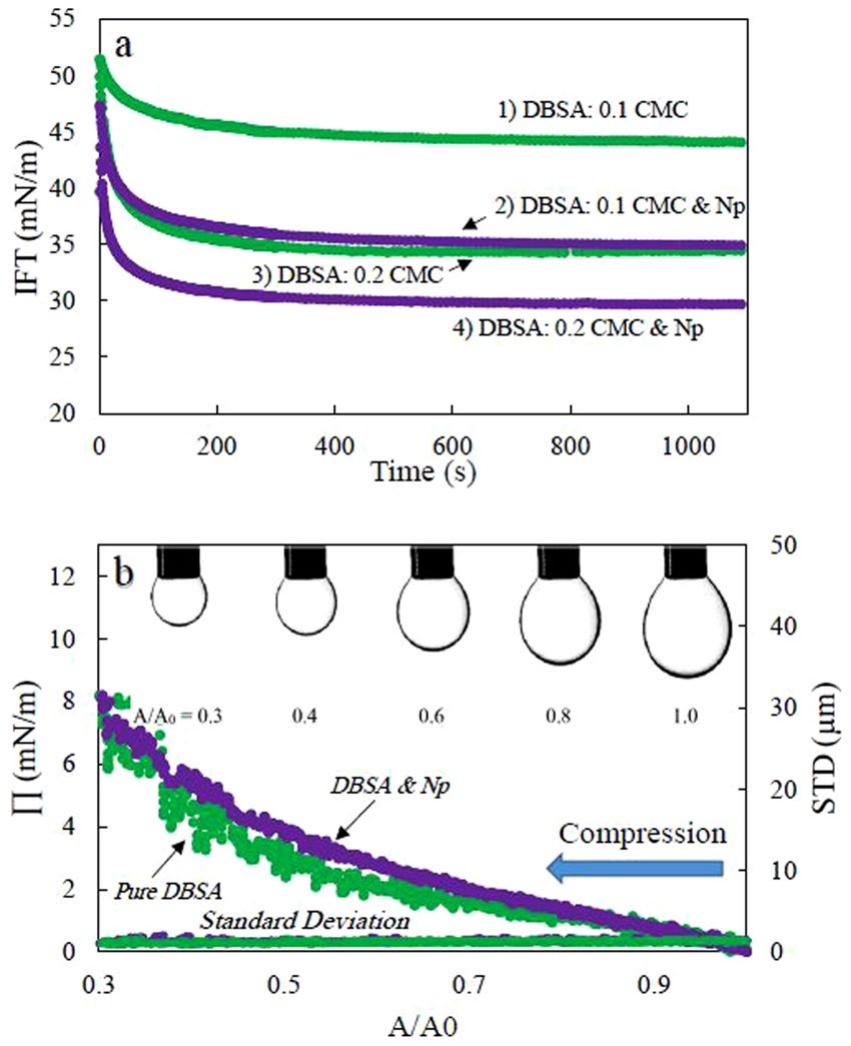
(**a**) The effect of nanoparticles (Levasil 300/30, 2.5 wt.%) on dynamic IFT of heptane/DBSA solution at two different surfactant concentrations. 1,3: pure DBSA, 2,4: DBSA & Np, (**b**) Variation of surface pressure and standard deviation of fitting the Laplace equation to drop profiles during drop compression of a DBSA solution (1.6 × 10^−5^ M: 0.1 CMC) in absence and presence of 2.5 wt.% nanoparticle (Levasil 300/30, 9 nm). Π: surface pressure, STD: the standard deviation of fitting the Laplace equation to the drop profiles, A/A0: the normalized surface area of droplet in respect to its initial value. No wrinkles appear at the drop surface during the compression process. The average error for IFT and surface pressure results is less than 0.7 mN/m and for standard deviation results is insignificant.

In the presence of silica nanoparticles (Levasil 300/30, 9 nm), the anionic surfactant of DBSA also behaves more efficient in reducing both the ST and IFT of the system. At the applied surfactant concentration (1.6 × 10^−5^ M: 0.1 CMC), the impact of surfactant on the surface tension has been insignificant (Fig. S5). However, in the presence of 2.5 wt% of nanoparticles, the surface tension has been reduced to about 67.5 mN/m at the end of the measurement time. Similar results can be seen for the water/heptane interface. At both concentrations of the used surfactant, 0.1 and 0.2 CMC, the IFT reduces more by adding silica nanoparticles. As shown in Fig. 8a, the further reductions in IFTs are about 10 and 5 mN/m for the surfactant solutions at a concentration of 0.1 and 0.2 CMC, respectively. These results also suggest that nanoparticles cause the surfactant to be more efficient and behave as if higher concentrations are used. According to the results, the equivalent surfactant concentration of the system containing 0.01 CMC DBSA and 2.5 wt.% Np is about 0.2 CMC; both systems show relatively similar behavior (Fig. 8a, curves 2 & 3).

Figure 8b represents the variation of surface pressure, Π, and the standard deviation of fitting the Laplace equation to drop profiles of two DBSA solutions during drop surface compression. The first system is without nanoparticles at a surfactant concentration of 1.6 × 10^−5^ M and the second one is the same surfactant solution mixed with 2.5 wt.% nanoparticle. As previously argued for SDS systems, both the surface pressure and the standard deviation variations are different from the cases where nanoparticles adsorb at the interface. These results support the impact of the repulsive interactions on increasing the surface activity of surfactant and the increased adsorption of the anionic surfactants to the interface in presence of negatively charged silica nanoparticles.

Despite many similarities in the surface behavior of the two anionic surfactants (SDS and DBSA) in presence of silica particles, there is one considerable difference in their dynamics of adsorption. It can be observed in Fig. 8a that the system containing 0.01 CMC DBSA and nanoparticles behaves quite similar to the DBSA-only solution at higher concentration (0.02 CMC) in terms of both, the dynamic and equilibrium IFT. However, as shown previously for SDS solutions (Fig. 4), when nanoparticles exist in the system, their dynamics of adsorption are much faster as compared to those with an equivalent surfactant concentration. Noting that SDS is known to hydrolyze in water^44–46^, the fast dynamics of adsorption for SDS solution in the presence of nanoparticles can be attributed to the interaction between nanoparticles and SDS-inherent impurities (mainly dodecanol) formed by hydrolysis. The interaction of silica nanoparticles with SDS inherent impurities will be thoroughly discussed in the following section.

### Interaction of nanoparticles with SDS inherent impurities

**Figure 9:**
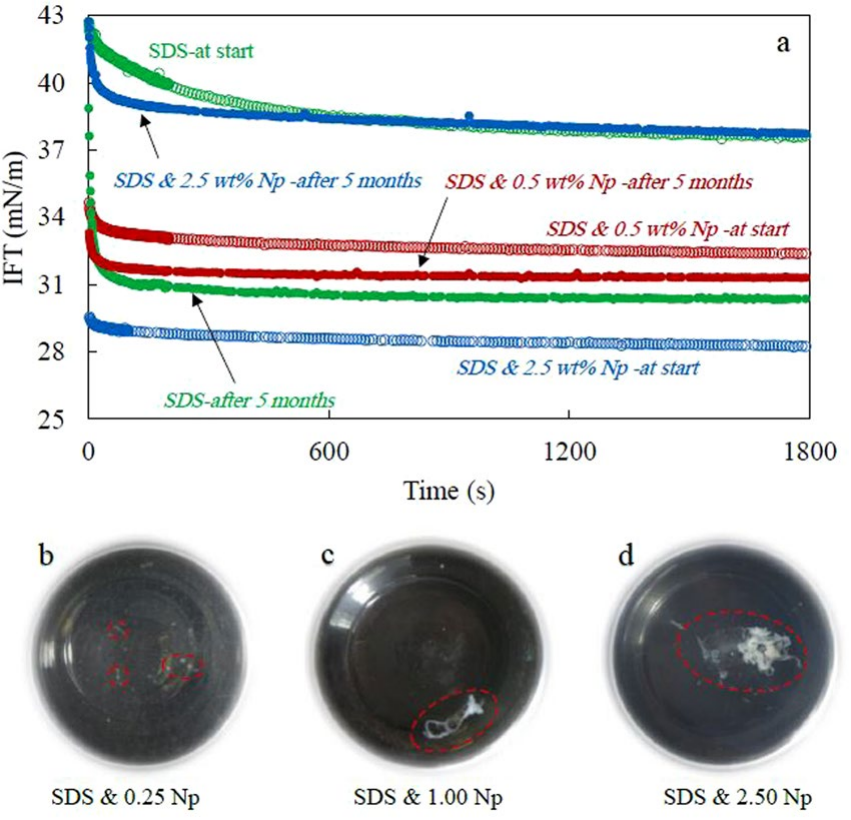
(**a**) Dynamic IFT of heptane/SDS solution mixed with different concentration of nanoparticles (Levasil 300/30, 9 nm) measured at two time-period, just after preparation of fluids (Empty points) and five months later (Filled points), (**b**–**d**) Bottles containing the aged solutions containing nanoparticles from the bottom view which show the formation of a white solid precipitate (nanoparticles-SDS impurity complexes).

To clarify the fast dynamics of adsorption in the presence of nanoparticles, the dynamic IFT of SDS solutions has been measured again five months after preparation of the solutions. The IFT measured for the fresh 0.05 CMC SDS solutions (with and without nanoparticles) and measured again for the solutions being for 5 months in the storage. The results are compared in Fig. 9a. As previously shown in the Fig. 1, when the measurements were performed just after preparation of the solutions, the equilibrium IFT values were lower in the presence of nanoparticles and it reduced more with increasing nanoparticle concentration. The dynamics of adsorption was also faster when nanoparticles were present in the system. However, it is completely reversed when the IFT has been measured after a relatively long aging time (5 months).

In this case, the lowest IFT is related to the SDS-only solution and the dynamics of adsorption has been faster for this solution. Its equilibrium IFT value is about 30.3 mN/m which is 7 mN/m lower than the equilibrium value of the freshly prepared solution. The lower IFT value of the aged SDS-only solution is related to the hydrolysis of SDS molecules to dodecanol in the water phase. Dodecanol is a highly surface-active component and as a second surfactant, its co-adsorption considerably affects the equilibrium IFT value. Furthermore, because of the SDS decomposition, the dynamics of adsorption can be also much faster than before. It is well known that at the applied concentration, the adsorption of SDS has a dynamics with a fast relaxation time which is about few seconds^47,48^. Therefore, it can be stated that the slow dynamics of adsorption observed in the freshly prepared solution is different from the SDS surface properties. It is just related to the existence of a minor amount of dodecanol molecules in the system which has been dramatically increased during a period of five months resulting from the SDS hydrolysis.

At the second measurement (aged solution), the IFT has a higher value for the systems containing nanoparticles in comparison to that for a surfactant-only solution. In addition, it increases further with increasing nanoparticles concentration. The IFT curve of the SDS solution with 0.5 wt.% nanoparticles did not change noticeably between the measurements at the two different times. The equilibrium IFT value decreased by less than one mN/m and the rate of its reduction was the same. However, for the SDS solution mixed with 2.5 wt.% of nanoparticles, the equilibrium IFT value raised by more than 9 mN/m.

These results refute the idea of accelerated SDS hydrolysis in presence of silica particles and the probable effects of the produced dodecanol on the observed IFT reduction in Fig. 1. In contrast, the results imply that silica nanoparticles alleviate the effects of dodecanol on the interfacial behavior. It seems that the surfactant contamination is depleted from the solution through the adsorption on the particles surface. Therefore, when the IFT has been measured shortly after the preparation of the solutions, the dynamics of adsorption is fast in the presence of nanoparticles. Obviously, it becomes faster with increasing nanoparticles concentration and consequently, increasing the available nanoparticles surfaces for adsorbing dodecanol. By aging the solution, more SDS molecules hydrolyze, the bulk SDS concentration decreases, and dodecanol concentration increases. The produced dodecanol adsorbs at the nanoparticles, and thus, the overall concentration of the surface-active components which affect the interfacial properties decreases. The adsorbed components on the nanoparticles’ surface can also screen the repulsive interactions between nanoparticles and SDS molecules. Therefore, logically, we must see higher IFT values at the later measurement time for system containing 2.5 wt.% nanoparticles.

As previously discussed, the relatively fast adsorption dynamics of both fresh (empty points) and aged solutions (filled points) indicates that the formed complexes of nanoparticle-SDS impurity (dodecanol) are not surface active and do not adsorb at the interface. This can also be inferred from the overall trends of interfacial elasticity (Fig. S3), the surface pressure, and the standard deviation of fitting the Laplace equation during the drop surface compression (Fig. 3). Another supporting evidence is the depletion and precipitation of the formed complexes, observed as white solid like precipitate. As we observed, the formation of the precipitate was continuous, and the amount of precipitation increased with the nanoparticle concentration (Fig. 9b–d). It should be noted that no precipitation was observed for nanoparticles-only solutions.

To sum up, it can be stated that the observed SDS interfacial behavior in the presence of nanoparticles can be attributed to two different effects of the nanoparticles: 1- the electrostatic repulsive interaction, leading to the increased surfactant surface activity and the lower IFT values, 2- the adsorption of the dodecanol on the surfaces of particles, affecting the adsorption dynamic and resulting in faster interfacial relaxation. It is worth mentioning that the hydrolysis of the surfactant is not the case for DBSA solutions. Therefore, the dynamic and equilibrium values of IFT of DBSA solutions with nanoparticles behaves quite similar to the pure surfactant system at respective higher concentrations.

## Materials and Methods

### Chemicals

Two types of commercial colloidal dispersions of silica nanoparticles including Levasil 300/30 and Levasil 100/45 (supplied by H.C. Starck GmbH & Co. KG Corporation) were used in this study. Silica nanoparticles in these dispersions are spherical and narrowly distributed around diameters of 9 and 30 nm, respectively^49–51^. The aqueous dispersions are free from any stabilizing additives and their high stability is obtained by a specific manufacturing process which provides large negative surface charge to the particles^52,53^. Two types of anionic surfactants, Sodium Dodecyl Sulfate (SDS, Merck, ≥99%) and Dodecyl Benzene Sulfonic Acid (DBSA, Behdash Chemical CO., ≥96%), were used without further purification. Sodium chloride (NaCl, Merck, ≥99%) was preliminarily heated for 24 h at 600 °C in order to remove possible surface active contaminants^54^. Deionized water and normal heptane (liquid chromatography grade, Merck, purity ≥99.3%) were utilized as the aqueous and oil phases, respectively. Heptane was passed through a chromatographic alumina column three times to remove impurities^55^.

### Preparation of surfactant-nanoparticle dispersions

Nanofluid dispersions containing twice the desirable concentration of silica nanoparticles were prepared by diluting the concentrated colloidal dispersions using pure water. The mixed particle-surfactant dispersions were prepared by adding the surfactant solutions with double target concentration to the diluted nanofluids. To avoid any particle aggregation during the dispersions preparation, the addition of surfactant solutions was performed drop by drop while continuously stirring the dispersions. In cases that the impacts of salt were also considered, the salt was first added to the surfactant solutions before mixing with nanoparticles dispersions. The final dispersions were sonicated in an ultra-sonic bath for 30 min and the experiments were performed immediately afterward to minimize the hydrolysis of SDS and its effect on the results.

### Measurements

The dynamic interfacial tension and dilational visco-elasticity (IFT and elasticity, hereafter) were measured using a drop profile analysis tensiometer (PAT1, Sinterface Technology, Germany). To this end, a pendant drop of the aqueous phase was created at the tip of a stainless steel capillary in a glass cuvette filled with pure heptane. The profile coordinates were extracted from the drop images and the IFT was measured by fitting the Gauss–Laplace equation to the experimental drop profile coordinates. The fully computer-controlled dosing system kept the drop surface/volume constant in the course of reaching the interface to its equilibrium adsorption.

The elasticity of interfacial layer was also evaluated by applying harmonic perturbations on the drop surface. In this way, the drop surface was subjected to sinusoidal oscillations and the response of the pre-equilibrated interface layer to its perturbations was analyzed using a Fourier transformation program. To remain in the range of a linear perturbation response, the frequency range and the amplitude of these perturbations were between 0.01 and 0.1 Hz and 8% of the initial drop surface area, respectively. The calculation procedure was described elsewhere in detail^56^. Moreover, the adsorption process of nanoparticle at the interface was evaluated by conducting high amplitudes interface compression. In this case, after the system reached the equilibrium IFT, the drop volume was undergone to a linear compression. Then, the variations of IFT and the deviation of the drop profile from its Laplacian shape were evaluated over the course of this process. The rate of compression and the amplitude of perturbation were 2.0 × 10^−5^ ml/s and about 70% of the initial interface area, respectively.

Zeta potentials of nanoparticles in aqueous nanofluid dispersions with varying surfactant/nanoparticle concentration and ionic strength were obtained using a Zetasizer ZEN3600 apparatus (Malvern Instruments, UK). All the experiments were conducted at 25 °C. All glassware and Teflon equipment in contact with the liquid phases during sample preparation or measurements were cleaned by concentrated sulfuric acid and washed carefully afterward with distilled water. The purity of the aqueous and oil phases and the absence of any contaminants in the systems were ensured by measuring the water/heptane dynamic IFT and elasticity before each measurement. All experiments were conducted at least three times to assure the repeatability of the results.

## Electronic supplementary material

Supporting information
