# Supplementary material for: The Role of Electrostatic Repulsion on Increasing Surface Activity of Anionic Surfactants in the Presence of Hydrophilic Silica Nanoparticles
Source: Sci Rep. 2018 May 8;8:7251. doi: 10.1038/s41598-018-25493-7 (PMC5940767; doi:10.1038/s41598-018-25493-7)
Supplement: Supplementary file 1 — Supporting information [file 41598_2018_25493_MOESM1_ESM.docx]

**Supporting information**

The Role of Electrostatic Repulsion on Increasing Surface Activity of Anionic Surfactants in the Presence of Hydrophilic Silica Nanoparticles

Hamid Vatanparast ^1,2^, Farshid Shahabi ^1^, Alireza Bahramian ^1^, Aliyar Javadi ^1^ & Reinhard Miller ^3^

^1^ Institute of Petroleum Engineering, College of Engineering, University of Tehran, Iran, ^2^ IOR Research Institute (IORI), Tehran, Iran, ^3^ Max-Planck-Institute for Colloid and Interface Science, D-14476 Golm, Germany. Correspondence and requests for materials should be addressed to H. V. (email: [h.vatanparast@ut.ac.ir](mailto:h.vatanparast@ut.ac.ir)) & A. B. (email: [abahram@ut.ac.ir](mailto:abahram@ut.ac.ir))

|  |
| --- |
| Fig. S1 Dynamic interfacial tension of n-heptane/water in the presence of nanoparticles (Levasil 300/30, 9 nm) with different concentrations, between 0.25 and 2.5 wt. %. The average error for each experiment is less than 0.5 mN/m. |

|  |
| --- |
| Fig. S2 Dilational elasticity of the equilibrium interfacial layer for n-heptane/water interface in presence of nanoparticles (Levasil 300/30, 9 nm) with different concentrations in the frequency range of perturbations between 0.01 and 0.1 Hz |

|  |
| --- |
| Fig. S3 Interfacial elasticity versus oscillation frequency for SDS solutions in the presence of nanoparticles (Levasil 300/30, 9 nm) with different concentrations. The amplitude of oscillation is 8% of the initial drop surface area and the surfactant concentration was fixed at 4.1 × 10^−1^ mM (0.05 CMC) in all experiments |

|  |
| --- |
| Fig. S4 Equivalent surfactant concentration as a function of nanoparticle concentration for composite systems at a fixed 0.41 mM (0.05 CMC) SDS concentration |

|  |
| --- |
| Fig. S5 Effect of nanoparticles (Levasil 300/30, 9 nm, 2.5 wt.%) on dynamic ST of a DBSA solution at a concentration of 1.6 × 10^−5^ M (0.1 CMC). The average error for each experiment is less than 0.5 mN/m. |
